# Digital genotyping of sorghum – a diverse plant species with a large repeat-rich genome

**DOI:** 10.1186/1471-2164-14-448

**Published:** 2013-07-05

**Authors:** Daryl T Morishige, Patricia E Klein, Josie L Hilley, Sayed Mohammad Ebrahim Sahraeian, Arun Sharma, John E Mullet

**Affiliations:** 1 Department of Biochemistry and Biophysics, Texas A&M University, College Station, TX 77843-2128, USA; 2 Department of Horticultural Sciences and Institute for Plant Genomics and Biotechnology, Texas A&M University, College Station, TX 77843, USA; 3 Department of Electrical Engineering, Texas A&M University, College Station, TX 77843, USA

**Keywords:** *Sorghum bicolor*, Grass, Genotyping, Polymorphism

## Abstract

**Background:**

Rapid acquisition of accurate genotyping information is essential for all genetic marker-based studies. For species with relatively small genomes, complete genome resequencing is a feasible approach for genotyping; however, for species with large and highly repetitive genomes, the acquisition of whole genome sequences for the purpose of genotyping is still relatively inefficient and too expensive to be carried out on a high-throughput basis. *Sorghum bicolor* is a C_4_ grass with a sequenced genome size of ~730 Mb, of which ~80% is highly repetitive. We have developed a restriction enzyme targeted genome resequencing method for genetic analysis, termed Digital Genotyping (DG), to be applied to sorghum and other grass species with large repeat-rich genomes.

**Results:**

DG templates are generated using one of three methylation sensitive restriction enzymes that recognize a nested set of 4, 6 or 8 bp GC-rich sequences, enabling varying depth of analysis and integration of results among assays. Variation in sequencing efficiency among DG markers was correlated with template GC-content and length. The expected DG allele sequence was obtained 97.3% of the time with a ratio of expected to alternative allele sequence acquisition of >20:1. A genetic map aligned to the sorghum genome sequence with an average resolution of 1.47 cM was constructed using 1,772 DG markers from 137 recombinant inbred lines. The DG map enhanced the detection of QTL for variation in plant height and precisely aligned QTL such as *Dw3* to underlying genes/alleles. Higher-resolution *Ngo*MIV-based DG haplotypes were used to trace the origin of DNA on SBI-06, spanning *Ma1* and *Dw2* from progenitors to BTx623 and IS3620C. DG marker analysis identified the correct location of two miss-assembled regions and located seven super contigs in the sorghum reference genome sequence.

**Conclusion:**

DG technology provides a cost-effective approach to rapidly generate accurate genotyping data in sorghum. Currently, data derived from DG are used for many marker-based analyses, including marker-assisted breeding, pedigree and QTL analysis, genetic map construction, map-based gene cloning and association studies. DG in combination with whole genome resequencing is dramatically accelerating all aspects of genetic analysis of sorghum, an important genetic reference for C_4_ grass species.

## Background

The acquisition of high quality genotyping information is essential for the assessment of genetic diversity, pedigree analysis, genetic map construction, QTL (Quantitative Trait Locus) analysis, marker-assisted breeding and association studies. Since development of RFLP (Restriction Fragment Length Polymorphism) technology [1], numerous methods for the analysis of DNA polymorphism have been developed including AFLPs (Amplified Fragment Length Polymorphism; [2]), SSRs (Simple Sequence Repeats), chip-based genotyping [2,3], GOOD [4], Taqman [5], TILLING (Targeting Induced Local Lesions in Genomes; [6]), SBE (Single-Base Extension; [7]) and SNPWave technologies [8]. Most DNA marker systems require the discovery and validation of SNPs (single nucleotide polymorphism) and INDELs (insertion- deletion) that are then targeted for high throughput assay. This approach allows the most informative and reproducible DNA markers to be utilized, but also can introduce ascertainment bias into analyses. Moreover, in diverse species with high rates of polymorphisms (>1/100 bp), indirect assays for DNA polymorphisms can be inefficient [9,10].

New high capacity DNA sequencing platforms provide an opportunity to transition from indirect assays of DNA polymorphism to genotyping by sequencing [9]. For many small genome species, complete genome resequencing is a feasible approach for genotyping. However, for species with large and highly repetitive genomes, the acquisition of whole genome sequences for the purpose of genotyping is inefficient and too expensive to be done on a routine basis. Moreover, most genotyping applications, such as marker-assisted breeding, require detection of only a subset of the extant genetic diversity among individuals to be effective. For these species and applications, targeted resequencing of specific sub-regions or ‘reduced representations’ of genomes provides sufficient information for genetic analysis.

Methods for acquiring ‘reduced representations’ of genomes for genotyping include the capture of DNA by hybridization to oligonucleotide arrays [11], skimming randomly sheared genomic DNA [12], and by using restriction enzymes [13]. The use of restriction enzymes for analysis of DNA polymorphism originated with RFLP analysis and is embedded in numerous DNA marker assays such as AFLP technology [2] and the related CRoPS (Complexity Reduction of Polymorphic Sequences) resequencing method for SNP discovery [13]. Baird and colleagues [14] successfully utilized resequencing of ‘restriction site associated DNA’ (RAD-tags) for SNP discovery and genetic mapping in stickleback species. A further increase in efficiency was achieved through the use of barcoding to enable multiplex sequencing of DNA pooled from numerous individuals. More recently, Genotyping-By-Sequencing (GBS) was described and tested on maize and barley, two grass species with large and highly repetitive genomes [10]. GBS utilizes multiplex sequencing of DNAs generated by a single restriction enzyme, *Ape*KI, a methylation insensitive restriction enzyme that recognizes the sequence GCWCG. One challenge noted in most of the prior genotyping-by-sequencing methods is variation in depth of sequencing among multiplexed samples, as well as site-to-site variation within the genome of a single genotype. This situation reduces efficiency and accuracy, requiring either greater depth of overall sequencing to obtain accurate information at a high portion of sites containing DNA polymorphisms, or indirect methods for haplotype reconstruction by imputation (e.g. [15]).

Our group is working on *Sorghum bicolor*, a diploid C_4_ grass that has a genome size of ~820 Mbp determined by flow cytometry [16] and encodes approximately ~30,000 genes, spanning ~150 Mbp of ‘gene space’ that is not highly methylated [17,18]. The remainder of the sorghum genome is largely composed of highly methylated repetitive DNA, preferentially localized in pericentromeric heterochromatic regions that have low rates of recombination [19]. Related members of sorghum can have much larger and more complex genomes that are more similar to polyploid grass species [20]. The sorghum genome sequence enables *in silico* testing of various genotyping by resequencing options, aids analysis of acquired sequences and the validation of results.

The sorghum research and public breeding community is small, therefore the development of chip-based methods for genotyping has been hindered by the start-up costs for this technology. Thus, we began developing a restriction enzyme guided genotyping-by-sequencing method termed Digital Genotyping (DG), when the 454 genome sequencing platform became available [21] and later transitioned this technology onto the Illumina GAIIx and HiSeq2000 to take advantage of their increased sequencing capacity [22]. DG was designed to enable analysis of sorghum genotypes at different levels of complexity (number of sites per genome), using a set of methylation sensitive restriction enzymes that have nested cut sites, so that information from all assays can be easily cross-referenced. Additionally, we report some of the reasons why variation in sequencing depth per site occurs within the same genome and how to minimize this source of inefficiency. Digital Genotyping was validated through genetic map reconstruction, QTL analysis, and haplotype/pedigree analysis.

## Results

### Template preparation and efficiency

The DG method provides an efficient means to produce accurate sequence-based genotype information for SNP discovery and genetic map construction within large populations in a short period of time. In the current iteration of the method using *Fse*I, index or barcode sequences incorporated into the adapters used for template synthesis permits combining DNA from up to 48 individual lines into a common pool for downstream processing. After sequencing on the Illumina GAIIx platform, the raw sequencing reads are processed and deconvoluted into individual groups by barcode. After parsing for reads containing proper bar codes and partial restriction site sequences, an efficiency of 80-90% is normally attained. Absence of a proper barcode and/or restriction site in a DG sequence is usually the result of improper purification of the products after the first ligation step or off-site PCR amplification. Initial DNA quality and accurate DNA quantitation also ensures higher yields of final useable sequence.

### Restriction enzyme selection

The restriction enzymes used for DG were selected based on six criteria: (1) sensitivity to DNA methylation to reduce template generation from repetitive regions of the sorghum genome; (2) GC-rich digestion sites that preferentially cut in or near genes; (3) lack or a limited number of sites of digestion in plastid DNA; (4) digestion at nested 4, 6, or 8 base restriction sites to enable varying depth of analysis; (5) generation of over-hanging termini that facilitate adapter ligation; and (6) presence of unique polymorphic sequences flanking restriction sites that provide good coverage of the genome. Restriction enzymes with nested 4, 6, and 8 bp recognition sites were sought so that information from analysis at different numbers of sites in the genome could be cross-referenced, enabling internal validation and coherent information sharing among different types of analysis (i.e., marker-assisted breeding, genetic maps, association studies). Restriction enzymes that met the criteria listed above were screened *in silico* to eliminate enzymes that cut preferentially in repetitive sequences and to confirm that sites of digestion would provide good coverage of the sorghum genome.

Several sets of restriction enzymes were identified that met our design criteria and after *in silico* analysis, *Fse*I (GGCCGG^v^CC), *Ngo*MIV (G^v^CCGGC) and *Hpa*II/*Msp*I (C^v^CGG) were selected for DG. These enzymes digest a nested set of GC-rich sequences that have *CCGG* as a common core recognition sequence. There are no *Fse*I restriction sites in the sorghum chloroplast genome, thereby eliminating potential background DG sequences derived from the plastid genome, present in >1,000 copies per cell in plant leaf tissue [23,24]. *In silico* analysis showed that these restriction enzymes would digest a non-methylated sorghum genome sequence at ~23,000, ~164,000, and ~1.4 M sites, generating two DG templates from each potential site of digestion (Table 1). *In silico* analysis identified 46,068 DG sequences adjacent to *Fse*I sites in the assembled reference genome sequence and 3,268 sequences in non-assembled super-contigs. One hundred sixty-six DG sequences in super-contigs were ‘unique’ and useful for DG marker analysis (data not shown).

**Table 1:** Restriction enzymes used for digital genotyping

**Restriction enzyme**	**Recognition sequence**	**No. of potential sequences RE sites x 2 ( *In silico *)**	**DG sequences unique, 33 bp ( *In silico *)**	**DG sequences unique, 33 bp (Sequenced >3x)**
***Fse*I**	GGCCGG^∨^CC	46,068	24,670	22,272
***Ngo*MIV**	G^∨^CCGGC	329,032	190,382	~155,000
***Hpa*II**	C^∨^CGG	2,872,516	~1,540,000	~572,000

Only unique DG sequences that were sequenced a minimum of three times and that mapped to a single location in the genome were used for genotyping. In this study, ‘unique’ DG sequences were defined as genomic sequences of a specified length adjacent to restriction enzyme recognition sites used for DG template preparation that mapped to either one location in the sorghum genome only or when mapped to more than one location, the top alignment differed from the second best alignment by at least 2 bp. The requirement for a two base difference among alignments was used so that a SNP allele in one DG sequence would not be confused with a DG sequence that maps to a different site. Most of the analysis presented here utilizes 33 bp of genomic DNA sequence for DG analysis. However, as DNA sequencing platforms have improved in accuracy, we have increased read lengths used for genotyping from 33 bp to 72 bp on the Illumina GAIIx and to 100 bp on the HiSeq2000. The number of 33 bp DG sequences in the sorghum genome obtained from BTx623 at a sequencing depth of 3x or greater ranged from 22,272 (*Fse*I) to ~572,000 (*Hpa*II), depending on the enzyme used to generate DG template (Table 1). Only 19,894 of the 22,272 unique *Fse*I DG sequences were used for genotyping. When two *Fse*I sites were located in close proximity and mapped to a unique overlapping genome sequence, only one of the DG sequences was used for analysis, thereby eliminating 2,378 sequences.

The substantial number of repetitive sequences adjacent to any set of restriction enzyme sites in the sorghum genome represents a potential source of inefficiency. For example, approximately 50% of the sequences flanking *Fse*I sites are repetitive. Repetitive DNA in plant genomes is highly methylated, therefore utilization of restriction enzymes sensitive to DNA methylation such as *Fse*I, *Ngo*MIV and *Hpa*II should reduce the acquisition of repetitive DG sequences. This expectation was confirmed. The ratio of unique/repetitive 33 bp DNA sequences obtained by sequencing DG templates generated using *Fse*I was ~6.3:1, compared to a ratio of 1:1 in the reference genome sequence determined by *in silico* analysis. Approximately 90% of the possible unique DG sequences flanking *Fse*I sites were represented in DG templates, indicating that ~5-10% of the *Fse*I sites flanked by a unique DG sequence were methylated. A small number of the *Fse*I derived DG sequences were also not identified due to the close proximity of two *Fse*I sites. Taken together, these results indicate that ~90% of the *Fse*I sites flanked by repetitive sequences were methylated and not represented in DG templates. A similar enrichment of unique sequences was obtained using the methylation sensitive restriction enzymes *Ngo*MIV and *Hpa*II in which approximately 81% and 37% of the *in silico* identified sequences, respectively, were actually sequenced. These data demonstrate that the use of methylation sensitive restriction enzymes significantly increases the efficiency of DG.

### DG marker discovery, frequency and distribution on chromosomes

When sequencing DG templates from different genotypes, the number of identified DNA polymorphisms will depend on several factors: (1) the number of ‘unique’ DG sequences derived from two or more genotypes that initially can be compared; (2) the length of the unique genome sequence acquired from DG templates, excluding the restriction enzyme partial site and barcode; and (3) the density of polymorphism among the genotypes analyzed in unique DG sequence space. If two parental lines used for genetic map construction have a polymorphism density of 1/500 bp in DG sequences, then analysis of 20,000 unique DG sequences 33 bp in length would be predicted to yield 1,320 DG markers for genetic analysis. This prediction was tested and the DG process further optimized through analysis of BTx623 and IS3620C, a pair of inbred sorghum genotypes, and 137 recombinant inbred lines (RILs) derived from these genotypes [25-27]. A precise alignment between the reference BTx623 genome sequence [18] and DG sequences derived from these genotypes were possible. To obtain sufficient information for analysis, an average of ~908,000 (+/− 278,200) sequences were obtained from DG templates prepared for each RIL. The templates were prepared and sequenced in pools of 24 RILs per lane on the Illumina GAIIx. The resulting range of sequencing depth per RIL varied from 335,000 to 1.9 M, indicating non-uniform pooling of DG templates from RILs constituting the pools. Approximately 88% of the reads acquired from the Illumina GAIIx contained the expected *Fse*I restriction site and barcode sequences and could therefore be used for further analysis.

We observed a sequencing error frequency of ~0.5-1% on the Illumina GAIIx. At this error rate up to one-third of the 33 bp DG sequences will contain sequencing errors. However, if the errors are random, the probability that the same error-containing sequence will occur several times in a specific DG sequence sequenced <100 times is low. To exclude this type of sequencing error, genetic analysis was based on DG sequences obtained three or more times from BTx623 or IS3620C.

**Figure 1:**
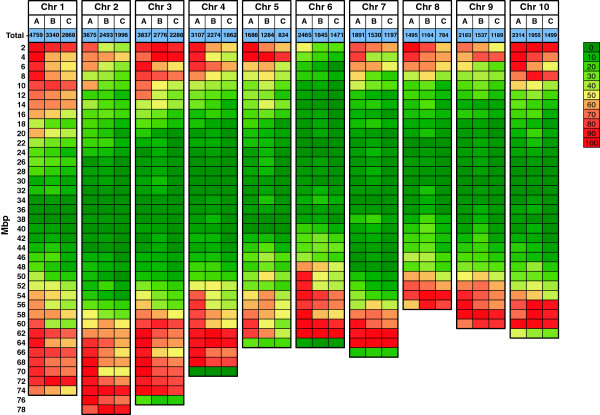
**Distribution of DG markers along the chromosomes of sorghum.** A heat map display of the distribution of **(A.)** predicted genes; **(B.)***Fse*I sites identified *in silico*; and **(C.)***Fse*I sites identified by sequencing along the ten sorghum chromosomes. For each 2 Mbp interval, values were determined as a percent of the total number of predicted genes or *Fse*I sites. For *Fse*I sites identified by sequencing, percentages were calculated based on the total number of *in silico* sequences.

There were 17,151 unique DG sequences obtained from both BTx623 and IS3620C that could be compared and searched for polymorphisms. This is less than the total number of unique DG sequences obtained from BTx623 (19,894) because DG sequences derived from one genotype can be missing in another genotype due to mutations in *Fse*I sites, differences in DNA methylation or missing data. While these sequences are a source of potential presence/absence markers, their utility was not further investigated. Alignment of DG sequences found in both genotypes identified 1,953 DG sequences containing a SNP or an INDEL that distinguish BTx623 and IS3620C. Overall, ~10% of the DG sequences compared were polymorphic, generating a predicted polymorphism rate of 1 SNP or INDEL per 289 bp. The putative DG markers spanned all ten chromosomes with higher density of DG markers per Mbp near the distal ends of chromosomes, where gene density is highest (Figure 1). Fewer DG markers/Mbp were present in the repeat-rich pericentromeric heterochromatic region of each chromosome, consistent with lower gene density, reduced rates of recombination, and higher levels of DNA methylation in these regions of the sorghum genome [19]. The general distribution of *Ngo*MIV markers was similar to those produced by *Fse*I, but at a higher density (data not shown). The largest physical gaps between DG markers, averaging 26 Mbp in size, occurred in the pericentromeric heterochromatic regions of each chromosome. Each of these physical gaps corresponded to 2 cM or less of the genetic map except for the pericentromeric region of LG-06 (Additional file 1) for reasons discussed further below.

### Genetic map construction with digital genotypes

**Figure 2:**
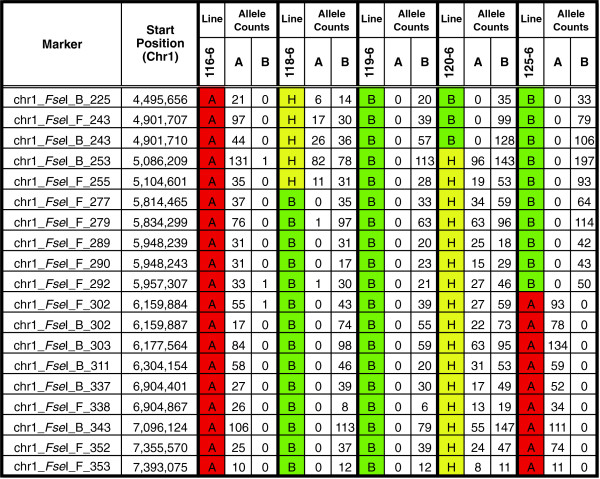
**Allele assignment with Digital Genotyping.** The raw DG allele counts and the physical order of the markers on chromosome 1 from 4.49 Mbp to 7.39 Mbp for a selected subset of five RILs are presented. Within this 2.89 Mbp interval, 19 polymorphic markers were identified that aligned to the reference sorghum genome. At each position the A-allele corresponds to BTx623; the B-allele corresponds to IS3620C; and the H-allele corresponds to a heterozygote.

DG marker genotypes were assigned initially using the following criteria: (1) DG markers sequenced less than 4 times from a RIL were marked as missing data; (2) if the ratio of allele sequences derived from a DG marker was > 4:1 then the genotype was scored as homozygous for the higher frequency allele sequence; and (3) if the ratio of allele sequences was 4:1 or less, then the genotype was scored as heterozygous. A total of 1,772 DG markers or ~89% of the 1,953 unique *Fse*I-derived polymorphic DG sequences from BTx623 and IS3620C were sequenced at sufficient depth in all 137 lines of the RIL population to enable high confidence analysis of DG marker segregation (<15% missing data per marker/137 RILs). The physical order of these markers was determined by alignment to the reference BTx623 genome sequence. An example of the raw data and the physical order of the markers on chromosome 1 for a subset of RILs can be found in Figure 2. Within this 2.89 Mbp interval of chromosome 1, 19 polymorphic markers were identified that aligned to the reference sorghum genome. The raw DG genotype data within this interval from five RILs is provided. At each position the A-allele corresponds to BTx623; the B-allele corresponds to IS3620C; and the H-allele corresponds to a heterozygote.

Overall, DG markers identified homozygous BTx623 genotypes ~50% of the time, homozygous IS3620C genotypes 45% of the time, and heterozygous genotypes at 5% of the loci. There were 894 redundant DG markers derived from either the same or a closely-linked restriction site that had identical segregation to a marker in the original dataset that were removed prior to genetic map construction (data not shown). The order of the non-redundant set of 878 markers was examined using Mapmaker/EXP ver. 3.0b [28] and 841 DG markers that could be ordered at LOD > 3.0 were used for genetic map construction. The order of the DG markers based on genetic analysis was identical to their predicted physical order across chromosomes based on alignment of marker sequences to the reference genome sequence with four exceptions (see below). The resulting genetic map spanned 1232.7 cM with an average resolution of 1.47 cM/marker (Table 2 and Additional file 1). The DG genetic map was similar in size but six-fold higher in marker resolution than a previous genetic map constructed using segregation information obtained from 477 SSR/RFLP markers, where data from 145 of these RFLP and SSR markers was used for genetic map construction [29].

**Table 2:** BTx623 x IS3620c recombinant inbred digital genotyping map statistics

	**Chr 1**	**Chr 2**	**Chr 3**	**Chr 4**	**Chr 5**	**Chr 6**	**Chr 7**	**Chr 8**	**Chr 9**	**Chr 10**	**Total**
**Chromosome Length (Mbp)**	73.84	77.93	74.44	68.03	62.35	62.21	64.34	55.46	59.64	60.98	659.23
**Marker Total**	139	102	127	92	43	64	76	55	58	85	841
**Total Genetic Distance (cM)**	166.6	152.2	147.7	131.4	105.3	113.6	105.4	96.0	112.3	102.2	1232.7
**Marker Density:** **1 marker / cM**	1.20	1.49	1.16	1.43	2.45	1.78	1.39	1.75	1.94	1.20	1.47
**1 marker / Kbp**	531.23	764.05	586.15	739.50	1450.05	972.01	846.61	1008.37	1028.20	717.43	783.86
**Largest interval (cM)**	6.9	7.4	5.5	10.0	8.1	39.2	7.0	7.3	11.2	6.4	

### DG genotyping accuracy and allele assignment

The fidelity of DG marker identification and the validity of the criteria for assigning genotypes were evaluated by comparing DG genotypes obtained from the 137 RILs to SSR genotypes previously collected from this population [29]. The location of DG markers on each chromosome was determined by alignment of DG sequences with the reference genome. SSR markers previously used for genetic map construction were also aligned to the genome sequence based on their oligonucleotide sequences and in an order consistent with prior genetic analysis [29]. The genotypes of 16 SSR markers on LG-01 and a DG marker located within 50 kbp of each SSR marker were compared in the 137 RILs. There was 99% agreement between genotypes assigned by the two types of markers in homozygous regions of the genome (data not shown). All but one of the 21 differences in genotype assignment out of 2,105 loci examined were due to SSR genotypes that interrupted haplotypes, possibly caused by double recombination events flanking these SSR markers, or more likely, due to genotyping errors associated with the SSR markers.

Genotyping accuracy was further quantified and criteria for assigning genotypes improved through analysis of pairs of DG markers derived from the same restriction site. Approximately 10% of the time, DG sequences flanking a common site of digestion were ‘unique’ and contained DNA sequence polymorphisms that distinguish BTx623 and IS3620C. Because the polymorphisms in these ‘pairs’ of DG markers are within 100 bp, genotypes assigned using data derived from the DG markers should be the same except in rare circumstances when recombination occurs within this interval. Therefore, the overall accuracy of DG genotype assignment was assessed by determining the consistency of the genotypes assigned by 40 pairs of these DG markers using data obtained from the 137 RILs. In homozygous regions of the RIL genomes, all 5,200 genotypes assigned using data from pairs of DG markers were identical (12 missing data points), indicating a high degree of genotyping accuracy in these regions of the RIL genomes (data not shown).

Criteria for assigning DG marker genotypes was further refined through analysis of sequences obtained from DG markers located in homozygous haplotypes. Only one allele sequence should be present in these regions of the RIL genomes. Therefore a count of the number of times the expected allele was sequenced compared to the alternative allele was used to estimate of the accuracy of genotype assignment based on DG marker data (average depth of sequencing/DG marker = 41). Overall, the expected DG allele was obtained 99.7% of the time (246 alternative allele sequences out of 76,032 sequences analyzed, data not shown). For 1,516 of the 1,728 DG markers analyzed, the correct allele was the only sequence obtained. One alternative allele was found in 183 DG marker sequences, two alternative alleles were found 25 times, three alternative alleles were found three times and four alternative alleles was found once. For DG markers where three or four alternative alleles were sequenced, the ratio of expected to unexpected allele sequence was >20:1. Thus, the assignment of DG genotypes in homozygous regions of a genome was very accurate.

Analysis of DG sequences from heterozygous (HET) regions of the RIL genomes (e.g. Figure 2) revealed that accurate assignment of DG genotypes in these regions is more challenging for several reasons. First, the two DG alleles from HET regions are sequenced on average only 50% as deeply as DG marker alleles from homozygous regions of the genome. Second, accurate assignment of HET genotypes requires more sequence reads per DG marker in order to be certain sufficient reads from both alleles have been acquired, if present, and an accurate ratio of allele sequences has been obtained. This potential source of false negative error can be reduced by greater depth of overall sequencing and by setting criteria that require more reads per DG marker for genotype assignment within heterozygous haplotypes. Third, pooled DNA from RIL progeny used for genotyping is occasionally enriched in alleles from one of the two parental genotypes due to non-uniform tissue pooling or progeny growth. This source of error can be minimized by pooling of equal amounts of tissue or DNA from large numbers of progeny. A fourth source of error occurs when alleles are sequenced with different efficiency (discussed below). A consideration of these factors and allele sequencing data described above led us to assign HET genotypes when both allele sequences corresponding to a DG marker are obtained three times or more and the ratio of allele sequences is <15:1. In addition, DG markers located within heterozygous haplotypes were only assigned genotypes when they are sequenced a total of 15 times or more. The criteria were independently assessed by carrying out DG analysis on F_1_ plants derived from a cross of Hegari x 100 M that would be expected have HET DG genotypes at all loci. Approximately 1,200 DG markers were analyzed and 99% of the genotypes called were HETs (data not shown). The remaining genotypes (A or B calls) occurred when too few reads were obtained from a given site (<10) and a few sites with highly skewed ratios of allele sequences (i.e. 26:1). We conclude that the empirical method developed here for identifying HET DG genotypes is reasonably accurate although further refinements will be possible in the future.

### DG-aided assembly of the sorghum reference genome

**Figure 3:**
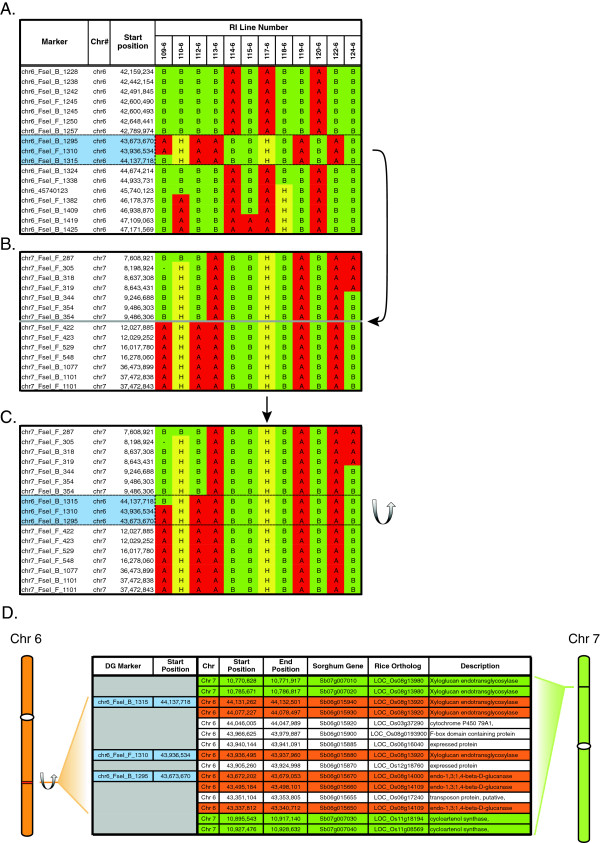
**DG-aided assembly of the reference sorghum genome sequence. (A.)** Physical location of DG markers chr6_FseI_B_1295, chr6_FseI_F_1310 and chr6_FseI_B_1315 on chromosome 6 and the related haplotypes generated from a selected subset of RIL lines in the region of miss-assembly. The genetic data within this interval do not demonstrate concordance with surrounding markers. Physical and genetic representation of chromosome 7 **(B.)** before and **(C.)** after insertion of the miss-assembled region from chromosome 6. The order of markers on chromosome 7 is reversed relative to their order on chromosome 6 to achieve concordance. **(D.)** Co-linearity of genes on sorghum chromosome 7 with orthologous genes on rice chromosome 8.

Inspection of DG haplotypes and analysis of DG marker segregation identified four instances where DG marker alignment to the reference genome sequence and the location of the DG marker in the genetic map was inconsistent. DG markers chr6_B_1315, chr6_F_1310, chr6_B_1295 were physically aligned to the BTx623 reference sequence on SBI-06 in the region spanning ~43.67-44.14 Mbp, however the haplotypes generated within this interval indicate that the markers and associated DNA are misplaced (Figure 3A). Genetic analysis placed all three markers in a cluster on SBI-07 between 9.49 and 12.03 Mbp (Figure 3B). Furthermore, a recombination event within the interval in one of the RI lines (109–6) indicates that the order of the markers is inverted, relative to their placement on SBI-06 (Figure 3C). Examination of the interval spanned by these three DG markers identified sorghum genes encoding xyloglucan endotransglycosylase/hydrase and cycloartenol synthase. These sorghum genes have the highest similarity to orthologous rice genes on rice chromosome 8. Rice chromosome 8 is collinear with SBI-07, specifically across the region where DG markers chr6_B_1315, chr6_F_1310, chr6_B_1295 mapped genetically (Figure 3D). Together, these results indicate that the sorghum genomic sequence, currently located on the SBI-06 pseudomolecule from approximately 43.67-44.14 Mbp is located on SBI-07 between 9.49-12.03 Mbp. One additional DG marker (DG marker chr2_F_2107) aligned to the reference genome on SBI-02 (66.31 Mbp), but this marker genetically mapped to SBI-03 between markers located at 9.87 and 10.05 Mbp (data not shown). This same DG marker was also genetically mapped to the same location on SBI-03 in a second RIL population (BTx642 x Tx7000; data not shown), indicating that DNA identified by this marker resides on SBI-03 instead of SBI-02. One explanation for these results is that the initial assembly of sequenced contigs into pseudomolecules representing the sorghum genome was not completely accurate, due to the large amount of repetitive DNA in the sorghum genome.

Super-contigs spanning a combined total of ~50 Mbp were not assembled into the pseudomolecules representing the 10 sorghum chromosomes, when the sorghum reference genome sequence was released [18]. Numerous DG sequences aligned uniquely with the non-assembled super-contigs and a subset of these contained polymorphisms that distinguished BTx623 and IS3620C (Table 3). Genetic analysis of the DG markers located in seven super-contigs allowed each to be ordered within the DG genetic map and placed in their approximate locations in the sorghum genome (Table 3).

**Table 3:** Genome coordinates of DG mapped super contigs

	**Super contig**		**Chromosome**	
**Super contig marker**	**Start positon (bp)**	**End position (bp)**	**LG**	**Coordinates (bp)**
super_12_*Fse*I_B_4	93,345	93,313	LG-01	41,820,858 - 44,466,741
super_12_*Fse*I_B_20	357,421	357,389	LG-01	
super_12_*Fse*I_F_48	1,098,822	1,098,854	LG-01	
super_12_*Fse*I_F_60	1,366,298	1,366,330	LG-01	
super_1919_*Fse*I_F_1	1,313	1,345	LG-01	51,294,508 - 51,595,270
super_916_*Fse*I_B_2	3,548	3,516	LG-01	57,928,418 - 58,404,926
super_2749_*Fse*I_F_1	507	539	LG-06	42,789,974 - 44,674,214
super_2273_*Fse*I_B_1	77	45	LG-07	60,223,905 - 60,421,993
super_2273_*Fse*I_B_2	828	796	LG-07	
super_514_*Fse*I_B_1	4,111	4,079	LG-08	49,298,803 - 49,486,219
super_337_*Fse*I_F_1	13,555	13,587	LG-09	0 - 1,083,522
super_337_*Fse*I_B_1	13,558	13,526	LG-09	

### DG enhanced QTL mapping

The utility of the DG genetic map for QTL analysis was investigated and compared to a prior study of QTL in the BTx623 x IS3620C RIL population [29]. Variation in plant height in the RIL population grown under field conditions in College Station, Texas was previously used to map QTL for this trait on SBI-03, SBI-06, SBI-07 and SBI-10 (Table 4). When phenotype data from the prior study was used in conjunction with the current DG map, the same four QTL for plant height were identified (Table 4). The height QTL on SBI-07 accounted for ~42% of the phenotypic variance and mapped coincident with *Dw3*, an MDR-type membrane transporter [30]. The LOD score for this QTL was ~25 based on the DG map and ~11.4 based on SSR/RFLP marker data used by Hart coworkers [29]. Because DG markers can be precisely aligned to the sorghum genome this allowed us to determine the physical location of the DG marker at the peak of the *Dw3* QTL locus. Marker chr7_B_1841 was located at 58.61 Mbp on SBI-07, perfectly aligned with the membrane transporter gene responsible for this QTL (Sb07g023730, located between 58,610,896 – 58,618,660 bp; Multani et al. 2003). Analysis of additive effects indicated that the *Dw*2 allele from BTx623 and the *Dw3* allele in IS3620C increased plant height consistent with previously assigned height genotypes of BTx623 (*dw1Dw2dw3dw4*) and IS3620C (*dw1dw2Dw3dw4*).

**Table 4:** Height QTL based on DG markers and phenotype data from Hart et al. (2001)

**Chromosome**	**Peak (cM)**	**Peak LOD**	**Additive**	**R2**	**Peak (bp)**	***Dw *locus**
3	6.1	5.78	10.8	0.0674	2,140,050	-
6	44.6	10.41	15.0976	0.1359	42,600,490	*Dw*2
7	77.5	25.16	−26.5685	0.4194	58,616,561	*Dw*3
10	49.2	4.74	9.637	0.0554	12,311,691	-

### DG haplotype and pedigree analysis

**Figure 4:**
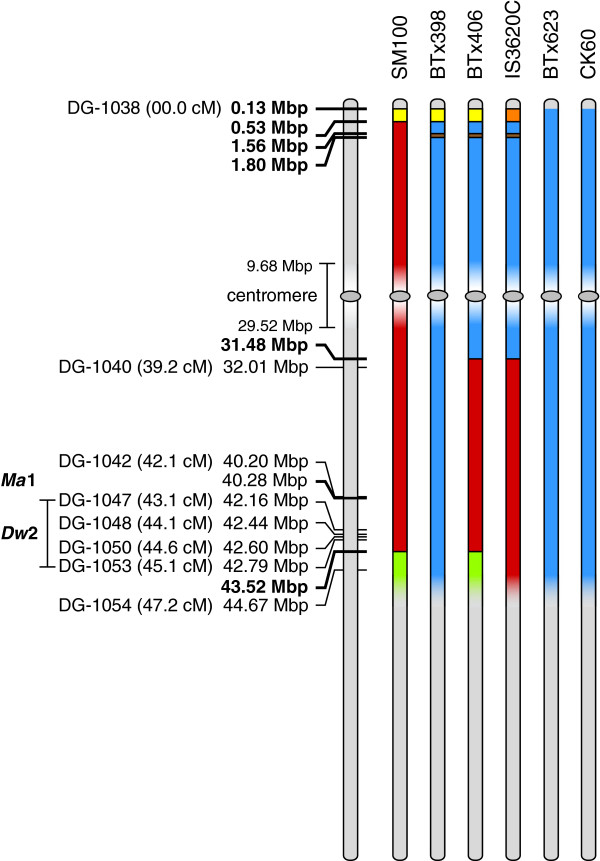
**DG Haplotype and Pedigree Analysis.** Graphical representation of chromosome 6 haplotypes determined for six sorghum accessions by DG analysis of *Fse*I and *Ngo*MIV markers. The region of chromosome 6 from 0.0 to 45.2 Mbp is represented. On the left, *Fse*I-derived DG markers with their corresponding genetic and physical locations are marked. For each *Fse*I DG marker an underlying *Ngo*MIV marker was also generated. Mbp values in bold represent haplotype block transitions determined by *Ngo*MIV markers. Details on additional intervening *Ngo*MIV markers can be found in Additional file 2. The approximate location of the centromere with the physical coordinates of the two closest *Ngo*MIV markers is shown. The physical location of *Ma*1 and genetic location of *Dw*2 are also shown. For the six sorghum accessions colored blocks represent haplotypes.

The pericentromeric region on SBI-06 is located close to the end of this chromosome (Figure 4). *Fse*I-derived DG markers immediately flanking the pericentromeric region [DG-1038 (chr6_F_1) at 12.9 kbp to DG-1040 (chr6_B_888) at 32 Mbp] spanned ~32 Mbp and 39.2 cM compared to < 5 cM for DG markers flanking the pericentromeric regions of the other sorghum chromosomes (Figure 4, Table 2 and Additional file 1). We hypothesized that part of this large gap in the genetic map might be due to introgression of DNA from BTx406, during conversion of IS3620 into the short, early flowering genotype IS3620C. *Ma1*, an important flowering time locus that controls photoperiod sensitivity [31], and *Dw2*, a locus that modulates stem internode length, are located adjacent to the pericentromeric region on the long arm of SBI-06, approximately 40–45 Mbp from the beginning of SBI-06. Sorghum accessions that are tall and late flowering are often converted to short, early flowering genotypes by crossing to BTx406 (*ma1, dw2*), followed by selection for short, early flowering plants [32]. Molecular introgression events that occurred during the generation of IS3620C were investigated by generating DG templates from BTx623, IS3620C and progenitors of these lines with *Ngo*MIV, a methylation sensitive restriction enzyme that recognizes the 6 bp sequence *G*^v^*CCGGC*. *Ngo*MIV generated 390 DG sequences that aligned to the genome in the interval from 0–45 Mbp of SBI-06. Data from 141 of the DG sequences spanned polymorphisms that distinguished the genotypes being analyzed (Figure 4 and Additional file 2).

A comparison of BTx623 and IS3620C DG genotypes showed that their genomes were highly polymorphic at the beginning of SBI-06 and from ~31.5 Mbp to the end of the long arm of SBI-06. However, DG sequences located between ~0.5 Mbp to ~31.5 Mbp showed a limited amount of polymorphism. The origin of this block of DNA in IS3620C was investigated by comparison with BTx406, the genotype used in the conversion program. The haplotype of the region of IS3620C from ~0.5-43 Mbp was nearly identical to BTx406, consistent with introgression of this block of DNA into IS3620 during the conversion process. BTx406 was derived from a cross of BTx398 and SA403 [32]. The haplotype of the region in BTx406 from 0.5-30.4 Mbp was identical to BTx398, indicating that during construction of BTx406, this genomic region was inherited from BTx398 (Figure 4). BTx398 and CK60, the immediate progenitor of BTx623 were developed during the period from 1920 to 1950 from a limited number of Kafir/Milo genotypes. BTx623 and CK60 have identical DG genotypes across this entire region of SBI-06, therefore, it is not surprising that the region from 0.5-30.4 Mbp of SBI-06 from BTx398, BTx406 and IS3620C is similar to BTx623. The low diversity of this region in IS3620C and BTx623 explains why there were so few DG markers in this portion of the genetic map derived from these lines.

The haplotype of IS3620C was nearly identical to BTx406 from ~32 Mbp to 43.5 Mbp of SBI-06, consistent with introgression of this region of BTx406 into IS3620C (Figure 4). A prior study showed that the recessive *dw2* allele in BTx406 was derived from Double Dwarf Yellow Milo, whereas the recessive *ma1* allele in BTx406 was derived from Early White Milo [32]. SM100, an early flowering (*ma1*) and short (*dw2*) genotype, was also derived from a cross of Double Dwarf Yellow Milo and Early White Milo [33]; therefore, the genotype of SM100 was compared to BTx406. The haplotype of SM100 from 31.5 Mbp to 45 Mbp was nearly identical to BTx406 and IS3620C, consistent with these regions being identical by descent and recessive for both *ma1* and *dw2*. The region of IS3620C from 45 Mbp to the end of SBI-06 was genetically distinct from BTx406, consistent with its origin from IS3620 (data not shown).

### Reducing variation in depth of DG marker sequencing

**Figure 5:**
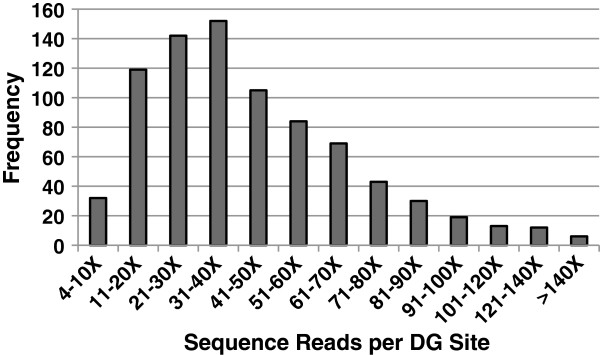
**Distribution of DG marker sequencing depth.** A histogram depicting the number of sequence reads obtained for each *Fse*I-derived DG site across the genome (*n* = 826) plotted against frequency.

Further enhancement of DG efficiency is possible if all DG markers could be sequenced at the same depth from each genotype analyzed. However, variation in depth of sequencing of different DG markers sequenced at least three times in any specific RIL varied > 40-fold (Figure 5). Importantly, the same DG markers were sequenced consistently at high or low relative frequencies from different RILs, indicating that variation in depth of sequencing was intrinsic to the DG template rather than a result of random variation or due to variation in template preparation. DNA templates with extreme base-composition bias, primarily high G/C-content, are sequenced with lower efficiency on the GAIIx platform. This bias is primarily a result of sub-stoichiometric generation of template by PCR [34]. Therefore, we examined the GC content of 300 bp adjacent to *Fse*I sites used to generate DG markers sequenced at high (> 80X) vs. low frequency (4-15X; Additional file 3). This analysis showed that DG markers sequenced at lower relative frequencies have higher GC content (~61.5%) and DG markers sequenced at higher frequencies have lower GC content (~44.6%). A similar conclusion was reached when comparing depth of sequencing and GC content of DG marker pairs derived from the same *Fse*I site (Additional file 3). In addition to overall GC content, there was variation in the relationship between the GC content of DG templates and depth of sequencing indicating that the sequence *per se*, in addition to overall GC content is probably affecting the efficiency of DG template generation and/or bridge amplification on the Illumina GAIIx (Additional file 3).

Variation in DG template length also affects the relative frequency of read acquisition on the Illumina GAIIx platform. The influence of template size on the relative sequencing efficiency was analyzed by generating templates with a wide range of fixed sizes using *Fse*I and *Mse*I, a methylation insensitive restriction enzyme that recognizes the four base sequence T^v^TAA. Barcoded adapters were ligated to *Fse*I-generated termini as usual, but the second adapter was ligated to the DNA termini created after digestion with *Mse*I instead of blunt-end termini generated by shearing. After sequencing on an Illumina GAIIx the full length of each sequenced DG template between the *Fse*I site and the nearest *Mse*I site was determined *in silico*. The relationship between template length and sequence frequency was determined. From this analysis it was evident that genomic sequences were obtained at very different frequencies from templates that ranged from 43 bp to 350 bp in length. Templates less than 65 bp were rarely sequenced because DNA purification during template generation preferentially removes small DNA fragments. Templates sequenced 40–72 times averaged 109 bp in length; templates sequenced 20–40 times averaged 148 bp; and templates sequenced 5–20 times were an average of 286 bp (data not shown). Reduced depth of sequencing of longer templates likely reflects a combination of decreased efficiency of amplification in PCR steps used in template preparation and less efficient bridge amplification of longer templates. These results indicate that a more uniform depth of sequencing among DG markers is achieved using randomly sheared DG templates that are within the same size distribution. To minimize read-depth variation due to template size, DNA from individual lines was pooled following ligation of barcoded adapters and sheared *en masse*, followed by ligation of the second adapter. DNA templates generated by shearing had an initial size range of 100–500 bp (data not shown). Templates of an optimal size for sequencing (~150-250 bp) were selected by sizing DNA on agarose gels, followed by excision and elution of DNA.

## Discussion

Digital Genotyping was developed to aid in the genetic analysis of sorghum and other grass species that have large repeat rich genomes. This general approach to genotyping is now feasible due to the rapidly decreasing cost of DNA sequencing over the past decade. Genotyping by resequencing is also efficient for species that lack array-based genotyping platforms because it combines polymorphism discovery and analysis and has the added benefit of reducing ascertainment bias. The acquisition of genotypes by sequencing is very accurate and rapid once template preparation is multiplexed using barcodes, informatics pipelines are established, and criteria for assigning genotypes have been validated.

### DG design features and accuracy

One of the central design principles embedded in DG is flexible depth of analysis and coherent cross-referencing of information derived from different applications. To accomplish this, we selected a set of restriction enzymes for DG template generation that recognize a nested set of 4, 6 or 8 bp sequences enabling analysis of ~520 K, 120 K and 20 K unique DG sequences, respectively, depending on the amount of information required. Further variation in depth of analysis and cost per assay can be obtained by changing sequence read length from 33 to 100 bp. This flexibility, combined with multiplex sample analysis, increases DG efficiency by allowing depth of analysis to be varied to match the information requirement of each application. For example, we currently multiplex 48 genotypes prepared with *Fse*I per lane on the Illumina GAIIx for genetic map construction and 12–24 genotypes per lane prepared with *Ngo*MIV for haplotyping, but use lower depth of analysis for marker-assisted breeding applications. With this design, *Fse*I-derived DG markers are a subset of the DG markers generated by *Ngo*MIV, and *Ngo*MIV DG markers are a subset of DG sequences derived from analysis with *Hpa*II, allowing coherent cross referencing between different levels of analysis. One important benefit of this transition has been greatly improved alignment and fine mapping of QTL relative to the underlying genes/alleles and precise inter-map alignment of QTL identified in populations derived from different parental genotypes.

The three restriction enzymes selected for DG have other useful properties including: (1) sites of digestion that are GC-rich and preferentially located in or near genes; (2) DNA methylation sensitivity that results in an ~6-fold enrichment of unique versus repetitive sequences; (3) lack of sites of digestion in plastid DNA for *Fse*I, eliminating a potential source of background due to the high copy number of the plastid genome; (4) generation of termini with overhangs that improve adapter ligation efficiency; and (5) high proportion of sequences flanking sites of digestion that are unique and polymorphic in sorghum germplasm. *In silico* analysis indicated that the distribution of restriction sites recognized by these enzymes would provide good coverage within the genic portion of chromosomes, a fact confirmed by sequencing DG templates. DG templates generated by restriction enzymes that are methylation sensitive have relatively low coverage in pericentromeric heterochromatic regions of sorghum chromosomes. These regions also have low rates of recombination. However, for some studies analysis of these regions using DG is of interest. This can be accomplished by preparing DG templates using restriction enzymes that are not sensitive to DNA methylation. For example, high-resolution DNA methylation mapping and analysis of pericentromeric heterochromatic regions of the sorghum genome can be carried out using the isoschizomers *Hpa*II (methylation sensitive) and *Msp*I (methylation insensitive).

DG was very accurate once parameters for allele identification and assignment of genotypes were optimized. During DG allele discovery most of the sequences containing random sequencing errors were eliminated with the requirement that DG sequences used for genetic analysis are obtained at least three times from parental lines. This criterion is useful during allele sequence discovery and eliminates most random sequencing errors that would occur when conducting DG analysis of species that lack reference genome sequences. DG markers, identified by comparing DG sequences derived from parental lines, genetically mapped with high fidelity to locations in the genome predicted by alignment of DG sequences to the reference genome. Comparison of genotypes assigned by pairs of DG markers derived from the same site of digestion confirmed the high level of accuracy of allele identification and genotype assignment in homozygous regions of RILs. An overall accuracy of allele identification of 99.7% was obtained. Genotype assignment in heterozygous regions was more challenging than in homozygous regions, requiring greater depth of analysis and more stringent criteria to assign genotypes accurately.

The overall efficiency of DG analysis has been enhanced by continuous improvements in sequencing, sample barcoding/multiplexing, and use of methylation sensitive restriction enzymes that access different numbers of DG sequences, depending on the information requirements of genotyping applications. On the other hand, the frequency of random sequencing errors on the Illumina GAIIx reduces efficiency by requiring a 20-40X average depth of sequencing and a minimum of 2-4X deep sequencing per DG marker during allele discovery and validation. However, following allele validation, lower depths of sequencing can be used to obtain high quality genotypes by designing informatics pipelines that preferentially search for validated allele sequences. We found that 99.7% of the time the expected DG allele sequence was obtained rather than the alternative allele. Overall, we routinely collect ~1,000 DG marker genotypes (15X average depth) from 400 samples per run on the GAIIx for ~ $8,000, excluding capital costs. Moreover, the estimated cost of genotyping is expected to be ~5-fold lower on the HiSeq2000 due to the increased number of templates sequenced per run and more uniform amplification of DG templates.

The main source of inefficiency in DG analysis identified in this study is variation in sequencing depth per genotype and among different DG markers. Variation in sequencing depth among multiplexed genotypes has been documented previously [10]. In the present analysis, the average depth of sequencing of individual genotypes that comprise pools of 24 RILs was 908 K (+/−278 K) but overall, sampling depth ranged from 335 K to 1.9 M. This results partly from variation in the amount of starting DNA from each genotype that is subjected to digestion and ligation of barcoded adapters in the first step of the protocol. In addition to careful quantitation of input genomic DNA, q-PCR could be used following digestion and ligation of bar-coded adapters to normalize template numbers derived from different genotypes at the template pooling stage [35]. Variation in template copy number among pooled genotypes can be compensated for by deeper sequencing, imputation of missing data, or by rerunning samples sequenced at low depth.

Variation in the relative depth of sequencing exhibited by different DG markers is a more significant issue. We found greater than 40-fold variation in sequencing depth for different DG markers from a given RIL and from pairs of DG markers derived from the same restriction site. The variation in the relative efficiency of sequencing among DG markers was consistently observed in different RILs and was associated with differences in the GC-content of DG templates. This result is consistent with the observation that DNA templates with high GC content are less efficiently bridge-amplified on the Illumina GAIIx leading to under representation of these sequences. New cluster generation kits that amplify templates of varying GC-content more uniformly for sequencing on the HiSeq2000 should reduce this source of variation.

Template length was another source of significant variation in relative efficiency of DG sequencing on the Illumina GAIIx. This was discovered when analyzing template sequences generated using *Fse*I and *Mse*I. Sequencing depth varied > 40-fold overall among templates of varying length regardless of GC content. For example, templates with an average length of 109 bp were sequenced 6-fold more frequently than templates that averaged 286 bp in length and DNA templates 350 bp or longer were rarely sequenced. This source of variation will be similarly present in template populations generated by *Ape*KI [10] or when using restriction enzymes such as *Hpa*II and *Mse*I as implemented in CRoPs technology [13]. Variation due to differences in template length can be overcome in part by greater depth of sequencing or by imputation of missing data [10,15]. However, this dynamic combined with variation in number of sequences/genotype in multiplexed samples could potentially result in significant amounts of missing data or overall loss of genotyping efficiency. This led us to utilize random shearing to generate DG templates of a more uniform size from all DG markers.

### DG utility and implementation

The utility of DG was tested and demonstrated through genetic map construction, improved genome sequence assembly, QTL mapping, and haplotype analysis. A DG genetic map was constructed by scoring 1,772 DG markers in 137 RILs derived from BTx623 and IS3620C. The resulting genetic map spanned 1,233 cM with an average resolution of 1.47 cM, a 6-fold improvement over a previous genetic map based on data from SSR/RFLP markers [29]. The DG map was used to reanalyze QTL for variation in plant height, using the original height phenotype values collected by Hart and coworkers [29]. The new QTL analysis identified the same four height QTL identified previously, but with higher LOD scores. More importantly, because DG map density is higher, and DG markers are located on the reference sequence, alignment between QTL and the underlying causative alleles is more precise. For example, the QTL peak corresponding to *Dw3* was aligned with the gene known to cause variation in height at this locus. Higher resolution *Ngo*MIV-depth DG haplotype analysis of IS3620C and BTx623 and their progenitors clarified the nature and origin of DNA present in SBI-06 in these genotypes. The analysis showed that IS3630C inherited DNA from approximately 0.5-32 Mbp from BTx398 via BTx406 during the conversion of IS3620 to a short, early flowering genotype. The common origin of BTx398 and BTx623 explained the low number of DG markers in the interval from 0.5 Mbp to 32 Mbp in the BTx623 x IS3620C genetic map. The results also confirmed that *ma1* and *dw2* in IS3620C traced back to recessive alleles present in BTx406, originally found in Milo genotypes as reported by Quinby [33].

DG analysis also helped improve the assembly of the sorghum reference genome sequence. DG marker mapping identified two regions of the reference sequence that were probably miss-assembled due to the high repeat content in these regions of the sorghum genome. Three DG markers that aligned to a region of the reference sequence on SBI-06 mapped in a cluster on SBI-07 and a DG marker aligned to the reference sequence on SBI-02 was mapped to SBI-03, indicating that the sequence assembly in these regions should be reexamined. Furthermore, DG markers aligned to sequences present in seven super-contigs that are not currently merged with the 10 pseudomolecules that comprise the reference sequence. Genetic analysis of these DG markers allowed the super-contigs to be placed in their approximate positions in the reference sequence. These results indicate that the construction of additional DG maps from other diverse parental genotypes will improve the quality and coverage of the sorghum reference sequence.

Our sorghum genomics and breeding group has transitioned to Digital Genotyping for nearly all genotyping applications. We utilize genotyping information derived from DG to quantify genetic relationships among accessions in the sorghum germplasm collection (n = ~40,000), for marker-assisted breeding and pedigree analysis, genetic map construction, QTL analysis, map-based gene cloning and association studies. Digital Genotyping in combination with whole genome resequencing is dramatically accelerating all aspects of genetic analysis of sorghum, an important genetic reference for C_4_ grass species.

## Conclusions

Digital Genotyping was developed to aid in the genetic analysis of sorghum and other grass species possessing large repeat-rich genomes. DG technology provides a cost-effective approach to rapidly generate highly accurate genotyping data. Our sorghum genomics and breeding group has transitioned to DG for nearly all genotyping applications. Restriction enzymes used for DG template generation recognize a nested set of 4, 6 and 8 bp restriction sites, providing a flexible depth of analysis and coherent cross-referencing of information derived from different genotyping applications. Currently, we utilize genotyping information derived from DG to quantify genetic relationships among accessions in the sorghum germplasm collection (n = ~40,000), for marker-assisted breeding, pedigree and QTL analysis, genetic map construction, map-based gene cloning and association studies. DG in combination with whole genome resequencing is dramatically accelerating all aspects of genetic analysis of sorghum, an important genetic reference for C_4_ grass species.

## Methods

### Plant material, growth conditions and DNA isolation

A collection of 137 F_6-8_ recombinant inbred lines (RILs) derived by single-seed descent from an initial cross between BTx623, an elite inbred line, and IS3620C, a converted inbred line highly divergent from BTx623, was used for genetic map construction [25-27].

Sorghum seeds were geminated in Sunshine MVP growing media (Sun Gro Horticulture) in a greenhouse for seven to ten days under normal sunlight supplemented with sodium halide lights. Temperatures varied from 24°C (night) to 30°C (day). Total genomic DNA was isolated from leaf tissue from 10–12 seedlings using a FastDNA Spin kit, according to the protocol provided by the manufacturer (MP Biochemicals). Purified genomic DNA was quantitated fluorometrically using a Qubit Fluorometer (Invitrogen).

### Index adapter design

**Figure 6:**
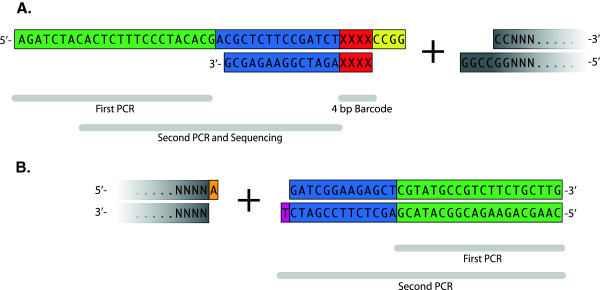
**Detailed view of *Fse *I and T-tailed adapters used during preparation of DG template for sequencing. (A.)** FseI and **(B.)** T-tailed adapter nucleotide sequences are shown. Primer binding sites for the first and second PCR and single-read sequencing reactions are indicated. Unique sequences within the adapter for the first and final PCR steps are denoted in green and blue, respectively. X’s denote the location of the 4 bp index sequence. The full complement of index sequences may be found in Additional file 4.

The nucleotide sequence for the Illumina Y-adapter (Oligonucleotide sequences © 2007–2012 Illumina, Inc. All rights reserved. Derivative works created by Illumina customers are authorized for use with Illumina instruments and products only. All other uses are strictly prohibited.), ligated to the opposing ends of DNA fragments, was used in the basic design of the index or barcode adapter and T-tailed adapters used for this study (Figure 6 and Additional file 4).

For the index adapter, additional bases were added to the 5′-end of the core Illumina Y-adapter sequence to facilitate two rounds of PCR. The PCR primers used in the initial and final amplification steps (see below) anneal to two unique regions within the adapter to reduce the potential for generation of amplification products from random sequences in the genome complementary to the PCR primers and not associated with an *Fse*I site. A unique four base pair index sequence is located immediately downstream from the sequencing primer binding site. Twenty-four unique index sequences were designed and incorporated into separate index adapters (Additional file 4). The index sequences were designed such that the *Fse*I site is not re-generated upon adapter ligation. A four base 3′-overhang sequence in the adapter immediately follows the index sequence to accommodate annealing to *Fse*I digested DNA. The same basic design was used for *Ngo*MIV index adapters except for addition of an *Ngo*MIV-specific four base 5′-overhang placed at the end of the adapter.

Adapter and PCR oligonucleotides (Additional file 4) were synthesized by Integrated DNA Technologies. For synthesis of adapters, oligonucleotides were resuspended to a final concentration of 100 μM in annealing buffer (10 mM Tris pH 8.0, 50 mM NaCl, 1 mM EDTA). Equal volumes of a complementary pair of oligonucleotides were combined. Annealing was accomplished by first heating the combined oligonucleotide solution to 94°C for 1 min and then allowing the mixture to slowly cool to room temperature (~1 hr). Freshly annealed adapters were diluted to a 5 μM final concentration for *Fse*I Index adapters and 25 μM for T-tailed adapters in annealing buffer. Oligonucleotides for PCR were diluted to 10 μM in 10 mM Tris pH 8.5.

### Digital genotyping template preparation

**Figure 7:**
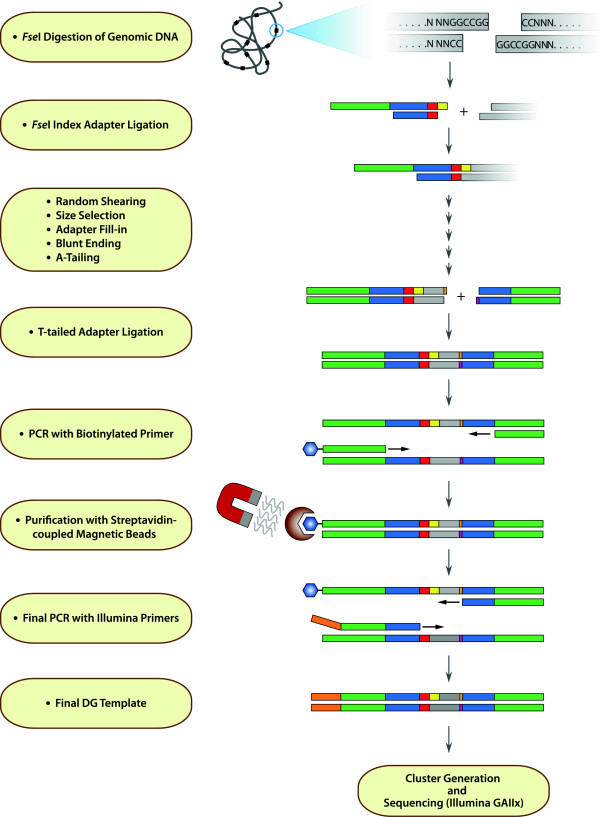
**DG template preparation workflow.** Genomic DNA is digested with *Fse*I. Index adapters are ligated to the *Fse*I ends. The DNA fragments are randomly sheared, followed by size selection on agarose gels. DNA fragments of a selected size are end-repaired. A T-tailed adapter is ligated to the repaired ends of the DNA. PCR is carried out with a biotinylated oligonucleotide primer complementary to the Index adapter. DNA fragments labelled with biotin are captured via magnetic beads. The purified DNA fragments are amplified by PCR with Illumina Primers. Amplification products are sequenced on the Illumina GAIIx sequencer. The colored regions in the DNA fragments correspond to the colored regions in the detailed views of the Index and T-tailed adapters in Figure 6.

The workflow used to prepare DG templates is shown in Figure 7. Genomic DNA from each individual line is arrayed in 96-well plates and digested with a restriction enzyme suitable for the intended analysis. For each individual sorghum line, 500 ng of total DNA was digested with 2 units *Fse*I in 20 μl reactions at 37°C for 2 to 4 hours, followed by heat inactivation at 65°C for 15 min. Indexed adapters were ligated to the *Fse*I sites by addition of 5 pmol index adapter and 1.5 units T4 DNA ligase directly to the digested DNA and incubated 4 hrs to overnight at 20°C. For pooling, up to 24 individual ligation reactions were combined. The pooled DNA was precipitated by addition of 1/10 volume of sodium acetate (pH 5.2) and 2 volumes of EtOH and incubating at −20°C for 30 min. The DNA was pelleted by centrifugation at 12,000xg for 10 min at 4°C. After washing with 70% EtOH, the DNA pellet was resuspended in 200 μl dH_2_O and transferred to 1.5 ml TPX microfuge tubes (Diagenode). The pooled DNA was sheared using a Bioruptor Plus (Model UCD-300; Diagenode) with 10 cycles of shearing on the LOW power setting with pulses of 15 sec ON/15 sec OFF at 4°C. The process was carried out for a total of four rounds (total elapsed pulse time 20 min) with a short centrifugation step between each round of shearing. Sheared DNA was purified and concentrated with AMPure XP beads, using the protocol provided by the manufacturer (Beckman Coulter Genomics). DNA fragments were separated on a 2% agarose gel with 0.2 μg/ml ethidium bromide. The gel and tank buffer also contained 2.0 mM guanosine (Fluka) to decrease potential damage to double-stranded DNA by ultraviolet light [36] during excision of gel bands. DNA fragments in the 150–250 bp range were excised from the gel and purified using a QIAquick Gel Purification kit (QIAGEN).

After size selection, a 3′-fill-in reaction of the 5′-overhang in the adapter was carried out in a reaction containing 20 units *Bst* DNA polymerase, large fragment and 200 μM each dNTP, at 65°C for 30 min. The DNA was purified using a QIAquick PCR purification kit and eluted in 40 μl EB buffer. DNA ends were blunt-ended using a quick blunting kit, following the directions provided by the manufacturer (New England Biolabs). DNA was purified using a QIAquick PCR purification kit and eluted in 35 μl EB buffer. Blunt-ended DNA fragments were A-tailed in a reaction containing 10 units Klenow polymerase, 3′-5′ exo^-,^ and 50 μM dATP at 37°C for 30 min. DNA was purified using a QIAquick PCR purification kit and eluted in 45 μl EB buffer. A T-tailed adapter was ligated to the DNA fragments by addition of 25 pmol index adapter and 3U T4 DNA ligase and incubated for 4 hours to overnight at 20°C. DNA was purified and concentrated with AMPure XP beads.

The resulting pool of DNA fragments contains a relatively small population of molecules with the two different adapters ligated to opposite ends of the genomic DNA fragments mixed with a much larger population of fragments with the second adapter ligated to both ends. To enrich for the former population of fragments, PCR was carried out (20 cycles) using Phusion DNA polymerase (New England Biolabs) with primers complementary to the two adapter sequences. The PCR primer complementary to the *Fse*I adapter sequence contains a biotin at its 5′-end. PCR reactions were purified using the QIAquick PCR purification kit.

Biotin-containing PCR products were isolated using streptavidin conjugated magnetic beads (Dynal M-280; Invitrogen). Briefly, ~2.0 μg PCR products in QIAGEN EB buffer were mixed with an equal volume of 2x Binding and Wash buffer (1x buffer: 5 mM Tris, pH7.5, 0.5 mM EDTA, 1.0 M NaCl). DNA was allowed to bind to the beads for 20 min with gentle shaking. After binding, beads were concentrated on a magnetic stand and the supernatant was discarded. Magnetic beads were washed with 300 μl 1x Binding and Wash buffer four times, followed by three washes with distilled water. To release the DNA fragments bound to the magnetic beads, the beads were washed once in 200 μl 2x SSC (1x: 0.15 M NaCl and 0.015 M Na Citrate) and resuspended in 50 μl 2x SSC. Beads were incubated at 95°C for 5 min. After collection of beads on a magnet, the supernatant was saved. The denaturation process was repeated and the supernatants were pooled. Single-stranded DNA was purified using the QIAquick PCR purification kit.

Single-stranded DNA products were used in a final PCR step (14 cycles) with primers containing sequences complementary to the flow cell, according to the protocol provided by Illumina. Amplified products were purified using a QIAquick PCR purification kit. The final products were quantitated by UV spectroscopy and diluted to 10 nM. The template was used in cluster generation (Single Read Cluster Generation Kit, version 4) and sequencing (36-cycle sequencing kit, version 4), according to standard Illumina protocols. Single-end sequencing was carried out for 38 cycles on an Illumina Genome Analyzer GAIIx.

For experiments utilizing the restriction enzymes *Ngo*MIV or *Mse*I, the index adapter or T-tailed adapter, respectively, was modifed with a 5′-overhang complementary to the sequence created by the specific restriction enzyme. All other downstream processing steps were identical to those used to synthesize *Fse*I-derived DG templates. Up to twelve individual lines were pooled for *Ngo*MIV-derived template.

### Informatic analysis of DG sequences

Base calling was performed using Illumina’s Real Time Analysis (RTA) software. Sequence text files were ge-nerated using GERALD in Illumina’s CASAVA v1.7 software package. The GERALD configuration file was set to trim 1 base at the 3′ end of each read since prephasing correction in CASAVA cannot be applied to the last base resulting in a slight increase in error at that position. Reads were then sorted into individual files by barcode, and filtered for 100% identity to the individual barcode plus partial restriction enzyme site. Identical reads were collapsed and read depth recorded and each sequence given a unique name that included the genotype from which it was generated and read depth using a series of custom python scripts. For genetic mapping with *Fse*I, unique sequences from the parental lines with a read depth of 3 or higher were aligned to the sorghum genome by BLASTN analysis using a Word size of 7 and match/mismatch scores of 2 and −3, respectively. The sorghum genome sequence was downloaded from http://ftp.jgi-psf.org/pub/JGI_data/phytozome/v6.0/Sbicolor/assembly/) and BLASTN performed on a local Linux workstation. After parsing the BLAST output files from the two parental lines, the results were manually inspected to remove sequences that aligned to two or more sites within the sorghum genome at the same e-value or percent identity. The output files from the parental lines were then combined and putative polymorphisms between the two identified using a custom python script. This script identified sequences from both parents that aligned at the same *Fse*I site within the genome and then performed pairwise comparison between the two to detect putative polymorphisms (SNPs and INDELs). To map putative polymorphisms identified between the two parents through the mapping population, a second python script was written that searched for each parental sequence from a given *Fse*I site in each progeny line and then recorded the appropriate parental allele (A, B or H) to produce a file suitable for input into either Mapmaker/EXP ver. 3.0b [28] or JoinMap V4.0 [37] for genetic map construction. The script was written to allow the user to specify the minimum read depth/sequence required to call an allele as well as the fold difference required to call a heterozygous loci in a line that contained both parental alleles at a given *Fse*I site.

For pedigree analysis with *Ngo*MIV template, following GERALD analysis and separation of the individual sequences into separate files based on the 4 bp barcode and partial RE site, the barcodes were trimmed from each sequence and the sequences uploaded to the CLC Genomics Workbench (CLC Bio) for sequence alignment and SNP detection. For alignment to the sorghum genome, the mismatch, insertion and deletion costs were set to 3 and sequences that matched more than one location identically were ignored. Using these alignment parameters, ~90% of the sequences generated from BTx623 aligned to the BTx623 sequenced genome. Following alignment, the CLC SNP Detection tool was used to identify potential SNPs in each genotype. Parameters for SNP detection included: a minimum read coverage at the potential SNP of 6, window length of 9, minimum average quality score of 15 and minimum central quality (i.e. quality of the SNP base) of 20. Once each genotype was processed using the SNP Detection Tool within the CLC Genomics Workbench, the individual files were exported in csv format and custom python scripts were used to combine the results and reformat the data for input into downstream analysis software (i.e. PowerMarker, STRUCTURE, FlapJack).

### Genetic map construction

The DG genetic linkage map was constructed using genotypes assigned by analysis of markers from 137 RILs derived from the BTx623 x IS3620C RI population [25-27]. Initial marker order was predicted based on alignment of DG marker sequences to the reference genome sequence [18]. Recombination frequencies of DG markers were determined using Mapmaker/EXP ver. 3.0b [28]. The command ‘map’ was used to calculate genetic distance between markers, using the Kosambi mapping function. When two or more DG markers mapped to identical locations, all but one of the markers were removed prior to the next step in mapping. The order of the remaining DG markers was confirmed using ‘order’ and ‘ripple’ functions. DG markers with LOD scores >3.0 were retained in the final DG genetic map.

QTL analysis was carried out using Windows QTL Cartographer version 2.5 [38]. Composite Interval mapping (model 6) was used for mapping QTLs. Threshold significance levels were determined by permutation analysis (1,000 permutations). Height measurements from Hart and coworkers [29] were used.

#### Data access

The sequences generated in this study have been deposited in the NCBI Sequence Read Archive (SRA) (http://www.ncbi.nlm.nih.gov/sra) under accession number [NCBI: SRX207965].

## Competing interests

The authors declare that they have no competing interests.

## Authors’ contributions

DTM conceived of the study, participated in its design, developed the method for generation of template, produced the material for sequencing, contributed to the interpretation of data and drafted the manuscript. PEK participated in the design of the study, performed the bioinformatic analysis, contributed to the interpretation of the data and helped to draft the manuscript. JLH participated in genetic map construction and QTL analysis. SMES wrote custom scripts used for bioinformatic analysis. AS participated in genetic map construction. JEM conceived of the study, participated in its design, contributed to the interpretation of data and drafted the manuscript. All authors read and approved the final manuscript.

## Supplementary Material

Additional file 1 **Title: BTx623 x IS3620C genetic map based on DG *Fse *I markers.** Description of data: A genetic map derived from the BTx623 x IS3620C recombinant inbred population was constructed using 841 ordered DG markers (LOD > 3.0). The genetic map covers 1232.7 cM with an average resolution of 1.47 cM/marker. For each chromosome individual DG markers and their recombination distances are presented. Click here for file

Additional file 2 **Title: Chromosome 6 haplotypes around the Dw2 region.** Description: Graphical representation of chromosome 6 haplotypes determined for six sorghum accessions by DG analysis of *Fse*I and *Ngo*MIV markers. A detailed view of all *Ngo*MIV markers is presented. The region of chromosome 6 from 0.0 to 45.2 Mbp is represented in the figure. For the six sorghum accessions colored blocks represent haplotypes. Click here for file

Additional file 3 **Title: Variation in depth of DG marker sequencing.** Description: The relationship between template counts and G/C-sequence composition was examined. The GC content of 300 bp adjacent to *Fse*I sites used to generate DG markers sequenced at high (>80×) vs. low frequency was determined. **(A.)** Twenty-five DG markers sequenced at relatively low frequency had an average G/C composition of ~61.5%. **(B.)** Twenty-five DG markers sequenced at relatively high frequency had an average G/C composition of ~44.6%. **(C.)** A comparison of depth of sequencing and GC content of DG marker pairs derived from the same *Fse*I site. Click here for file

Additional file 4 **Title: Adapter and PCR oligonucleotide sequences.** Description: A tabular list of all Adapter and PCR oligonucleotide sequences used to prepare DG template. Barcode sequences used in the *Fse*I and *Ngo*MIV adapters are also presented. Click here for file
