# Supplementary figures and images for: Digital genotyping of sorghum – a diverse plant species with a large repeat-rich genome
Source: BMC Genomics. 2013 Jul 5;14:448. doi: 10.1186/1471-2164-14-448 (PMC3716661; doi:10.1186/1471-2164-14-448)

# Additional File 1 - BTx623 x IS3620C RIL Genetic Map

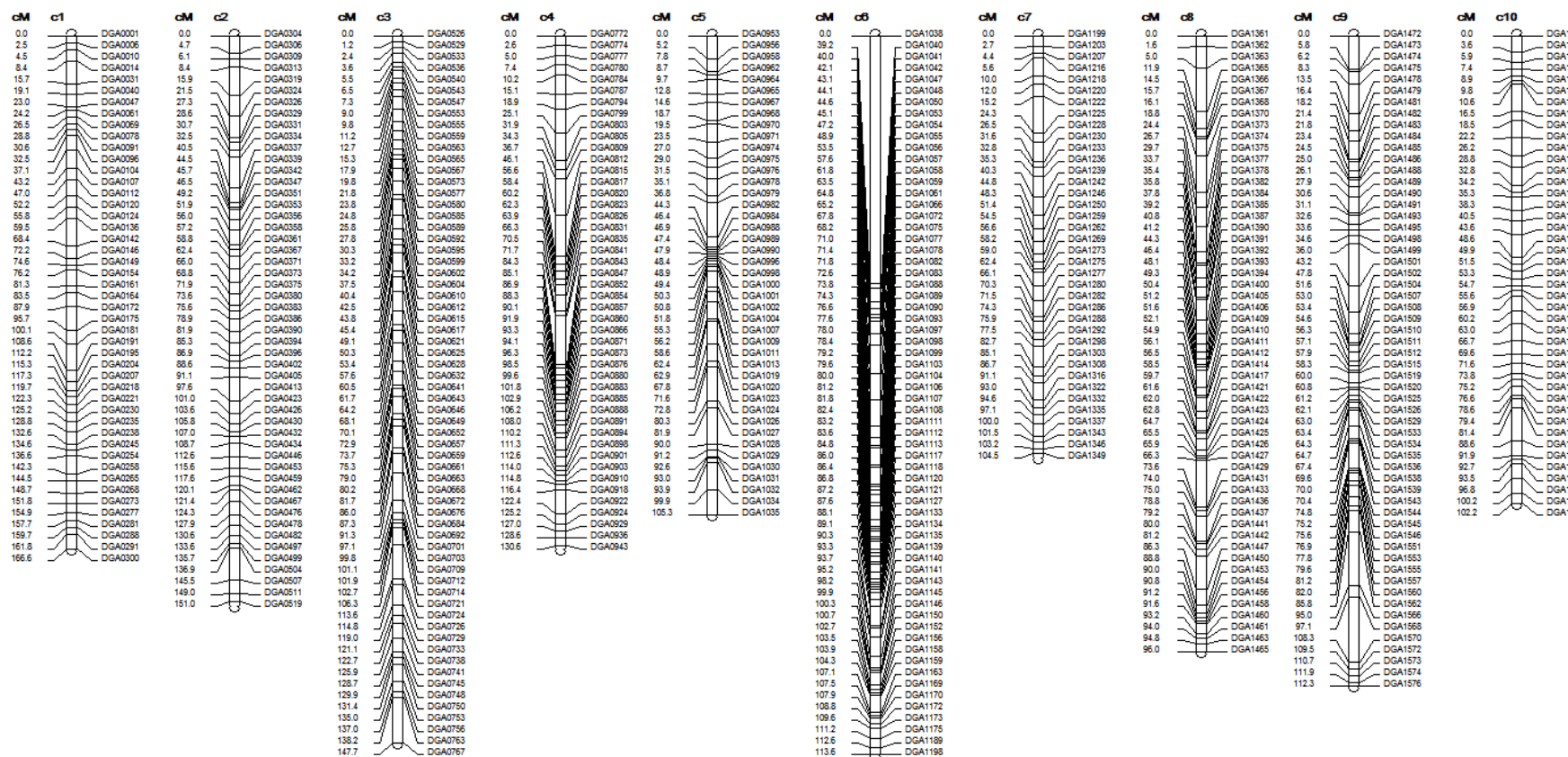

Supplement: Additional file 1 — Title: BTx623 x IS3620C genetic map based on DG Fse I markers. Description of data: A genetic map derived from the BTx623 x IS3620C recombinant inbred population was constructed using 841 ordered DG markers (LOD > 3.0). The genetic map covers 1232.7 cM with an average resolution of 1.47 cM/marker. For each chromosome individual DG markers and their recombination distances are presented. [file 1471-2164-14-448-S1.pdf]
